# Systematic Review of Intrapartum Fetal Heart Rate Spectral Analysis and an Application in the Detection of Fetal Acidemia

**DOI:** 10.3389/fped.2021.661400

**Published:** 2021-08-02

**Authors:** Luísa Castro, Maria Loureiro, Teresa S. Henriques, Inês Nunes

**Affiliations:** ^1^Faculty of Medicine, Centre for Health Technology and Services Research (CINTESIS), University of Porto, Porto, Portugal; ^2^Health Information and Decision Sciences Department - MEDCIDS, Faculty of Medicine, University of Porto, Porto, Portugal; ^3^School of Health of the Polytechnic of Porto, Porto, Portugal; ^4^Faculty of Engineering, University of Porto, Porto, Portugal; ^5^Institute of Biomedical Sciences Abel Salazar, University of Porto, Porto, Portugal; ^6^Centro Materno-Infantil do Norte – Centro Hospitalar e Universitário do Porto, Porto, Portugal

**Keywords:** fetal acidemia, fetal heart rate, spectral analysis, frequency bands, intrapartum

## Abstract

It is fundamental to diagnose fetal acidemia as early as possible, allowing adequate obstetrical interventions to prevent brain damage or perinatal death. The visual analysis of cardiotocography traces has been complemented by computerized methods in order to overcome some of its limitations in the screening of fetal hypoxia/acidemia. Spectral analysis has been proposed by several studies exploring fetal heart rate recordings while referring to a great variety of frequency bands for integrating the power spectrum. In this paper, the main goal was to systematically review the spectral bands reported in intrapartum fetal heart rate studies and to evaluate their performance in detecting fetal acidemia/hypoxia. A total of 176 articles were reviewed, from MEDLINE, and 26 were included for the extraction of frequency bands and other relevant methodological information. An open-access fetal heart rate database was used, with recordings of the last half an hour of labor of 246 fetuses. Four different umbilical artery pH cutoffs were considered for fetuses' classification into acidemic or non-acidemic: 7.05, 7.10, 7.15, and 7.20. The area under the receiver operating characteristic curve (AUROC) was used to quantify the frequency bands' ability to distinguish acidemic fetuses. Bands referring to low frequencies, mainly associated with neural sympathetic activity, were the best at detecting acidemic fetuses, with the more severe definition (pH ≤ 7.05) attaining the highest values for the AUROC. This study shows that the power spectrum analysis of the fetal heart rate is a simple and powerful tool that may become an adjunctive method to CTG, helping healthcare professionals to accurately identify fetuses at risk of intrapartum hypoxia and to implement timely obstetrical interventions to reduce the incidence of related adverse perinatal outcomes.

## Introduction

The fetus depends on the mother for oxygen and carbon dioxide exchange and for glucose supply, through the placenta, to maintain aerobic metabolism and adequate energy production. If there is a problem in maternal circulation, maternal respiratory function, placental blood supply, blood gas exchange within the placenta, or fetal and umbilical circulation, the fetus may suffer hypoxia. This decreased oxygen concentration in fetal arterial blood—hypoxemia—can lead to decreased oxygen in the tissues—hypoxia/metabolic acidosis—which, if not reversed, may cause a series of long-term sequelae, such as hypoxic-ischemic encephalopathy – (HIE) leading to the later development of cerebral palsy, or perinatal death (1). In order to prevent brain damage or perinatal death, it is important to diagnose hypoxia as soon as possible, allowing obstetrical interventions to reverse it before irreparable damage develops. To make this possible and practical to screen for, intrapartum fetal monitoring with cardiotocography (CTG), a technique which evaluates fetal heart rate (FHR) and uterine contractions, has been widely used to help detect fetal hypoxia/acidosis and prevent related adverse perinatal outcomes (2).

FHR signals can be extracted using external or internal methods. The most common mode of acquisition in clinical practice is non-invasive and consists of using a Doppler probe applied on the maternal abdomen. In some situations, invasive FHR acquisition through an electrode placed on the fetal scalp (FSE), using fetal electrocardiogram, is preferred, enabling a more effective signal acquisition, when a satisfactory trace cannot be obtained by external FHR, for example, due to maternal obesity. FSE can only be used during labor, after the rupture of membranes and the beginning of cervical dilatation (3). Although not yet fully available in clinical practice, another promising technique is the transabdominal electrocardiogram which is more reliable than the ultrasound to acquire FHR signals, but still having some limitations in separating the fetal from the maternal heart rate signal. At last, fetal magnetocardiography (fMCG), the magnetic analog of fetal ECG, used in research settings and a highly effective method to evaluate fetal arrhythmias, uses the complexity of Superconducting Quantum Interference Device (SQUID) technology and is very expensive, therefore not being suitable for clinical routine use (4).

CTG visual analysis has limitations regarding its validity, reproducibility, and inter and intra-observer agreement due to the complex nature of FHR traces (5, 6). In addition, it can sometimes lead to unnecessary obstetrical interventions, such as cesarean section and operative vaginal delivery, which are associated with maternal and perinatal risks (7–9). To tackle these problems, attempts have been made to analyze CTGs with computerized systems, automating the diagnosis of fetal hypoxia/acidosis (10). Linear and non-linear algorithms have been applied to FHR signals aiming at improving performance results in the prediction of acidemia (11–13). However, although promising results have been achieved with non-statistical and complex indices (14–16), only statistical tools have been included in clinical practice, probably enabling a more intuitive interpretation by the practicing obstetrician. A crucial feature of the FHR traces is variability, which is physiologically related to the fetal heart autonomous nervous system control (17). When compared to statistical indices, spectral analysis has been referred to as providing a more appropriate evaluation of the FHR non-linear periodic changes (18). As such, spectral analysis of the FHR has been extensively explored for the detection of several pathological conditions in the fetus (19–23).

Spectral analysis determines the energy in specific frequency components of heart rate variability, and changes in the power distribution of the FHR have been considered as a predictor of fetal distress in antepartum and intrapartum settings, reflecting the autonomic nervous system (ANS) activity (24–26), which is activated by hypoxemia (25, 27). Parasympathetic and sympathetic systems compose the two divisions of the ANS, which regulates the body's unconscious actions. While the parasympathetic system acts in *rest-and-digest* and *feed-and-breed* situations, the sympathetic system acts in *fight-or-flight* situations. This method takes into consideration various frequency domains that cause complex heart rate changes and are related to diverse systems of the human body (28). Although several promising results were observed in various studies using this technique, comparison problems arise when different authors use a disparate selection of frequency bands to integrate the spectrum (24).

Several methods have been proposed for power spectral estimation of real time series, which basically fall into two groups: non-parametric techniques such as the periodogram and the Welch method (modified periodogram), and parametric techniques which identify a model representing a good approximation of the sampled signal, such as autoregressive models. Autoregressive models are defined by some order, and their coefficients can be estimated by the least-squares procedure or through the Yule-Walker equations. While all spectral methods provide only estimates of the real power spectral density (PSD) of the signal and comparable results, non-parametric methods have the advantages of simplifying the algorithm and its high processing speed. On the other hand, parametric methods provide accurate PSD estimation even on a small number of samples but require the verification of the selected model's suitability and its order or complexity (29, 30).

It is known that in the FHR power spectrum, one observes four regions, different from the components observed in the power spectrum of an adult heart rate signal (17, 29). These four components in the spectrum of the FHR have been proposed in 2003 by Signorini et al. (17) and adopted by several studies (3, 31–35). The lowest is the very low frequency (VLF), ranging from 0 to 0.03 Hz, being related to long period events and non-linear contributions; followed by the low frequency (LF), ranging from 0.03 to 0.15 Hz and being mainly associated with neural sympathetic activity. These two low frequency bands, VLF and LF, are also associated with clinical variability such as accelerations and decelerations. The next is a typical band of the FHR spectrum, the movement frequency (MF), located between 0.15 and 0.5 Hz, marking maternal breathing and fetal movements (36, 37). The high frequency (HF) ranges from 0.5 to 1 Hz and indicates the presence of fetal breathing. Other parameters are recommended besides these specific spectral power bands, such as FHR mean and variance values and the LF/(HF + MF) ratio. This ratio quantifies the autonomic balance between neural control mechanisms from sympathetic and parasympathetic origins (17), in parallel with the ratio formulated as LF/HF, normally calculated in adults (29).

Thereby, the primary goal of this work was to determine which frequency bands are the best at screening fetal hypoxia/acidosis. First, a systematic review of the literature was conducted to collect all the different frequency domain bands used for FHR spectral analysis. Then, the power of the different bands found was computed on an open-access dataset using non-parametric spectral analysis in order to address their performance at screening fetal hypoxia/ acidosis.

## Materials and Methods

### Systematic Review of FHR Spectral Analysis

A systematic review was conducted in order to obtain a collection of the original papers reporting FHR spectral analysis for revision of the spectral bands employed. All original research articles reporting spectral power analysis of real human fetal heart rate (or RR intervals) signals, published until the end of June 2020, and available in PubMed, were included in the analysis. For this purpose, papers were obtained from an online search on PubMed/Medline with the following query:

(“spectral”[All Fields] OR “spectrally”[All Fields] OR “frequency domain”[All Fields] OR “frequency analysis”[All Fields] OR “frequency parameters”[All Fields] OR “linear models”[All Fields])

AND “fetal heart rate”[All Fields]) AND (“human s”[All Fields] OR “humans”[MeSH Terms] OR “humans”[All Fields] OR “human”[All Fields]).

From the set of papers obtained with the query described, two researchers, familiar with signal analysis, selected the papers to include. This selection was based on the title and abstract only, and performed independently, by each researcher. The papers to include were then distributed randomly to three of the authors who extracted, from the full paper, the relevant information for this study. During full revision of the studies, referenced papers, which were not captured by the query, were also manually selected. Previous studies have shown that the distribution of the frequency spectrum suffers changes with the baby maturation (38), hence the revision was limited to articles reporting analysis of signals referring to the term of pregnancy and to the intrapartum period. The exclusion criteria were: language other than English; no report of signal analysis (mainly reviews); reported analysis in simulated signals or from non-human fetuses; reported analysis on signals obtained from magnetocardiography (by its lack of applicability in clinical practice); and used data from the antepartum period. In the end, a consensus was reached about the papers that accurately reported spectral analysis of intrapartum FHR and that were included in the final revision. The information extracted from the studies, beyond the frequency bands used, included, if available, the employed spectral analysis method, the interpretation of the bands used, the type of signal spectrally analyzed (FHR or RR intervals) and its sampling frequency, and the signal acquisition mode.

**Figure 1:**
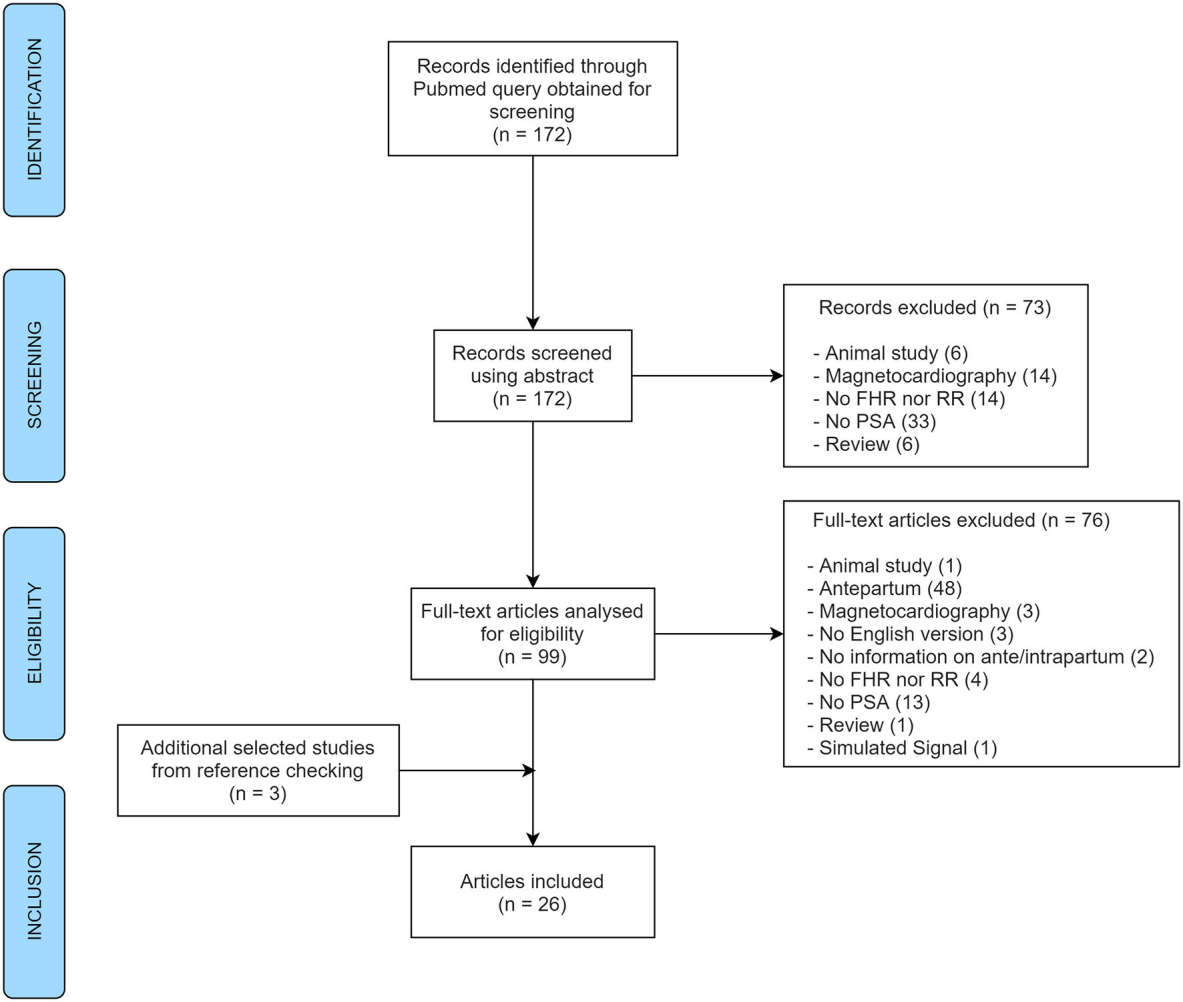
PRISMA flow diagram of the article selection process for the systematic review (FHR, fetal heart rate; RR, interval between R peaks; PSA, power spectral analysis).

A total of 172 abstracts were identified for screening, selected automatically by the query, and 3 additional articles were obtained from reference checking during the eligibility step. After exclusions, 26 articles remained to be included in the review (Figure 1). This systematic review focused on the frequency spectral bands being used and reported for spectral analysis of FHR in the literature. It was not in the scope of this research to consider other information from those articles, commonly addressed in systematic reviews such as main outcomes, discriminatory validity or other results. In this research, the information retrieved from the reviewed studies concerned only the methodology regarding the spectral bands used.

### Application in Fetal Acidemia Detection

#### FHR Data

In order to compare the performance of the various spectral indices used in the articles reviewed, the first open-access database for research analysis in intrapartum CTG was used, enabling the objective comparison between results from different methods (39). The open-access database CTU-UHB consists of a total of 552 intrapartum FHR recordings obtained, between April 2010 and August 2012, at the labor ward of the University Hospital in Brno, Czech Republic. Along with the intrapartum CTGs, relevant fetal (including biochemical parameters of umbilical arterial blood samples) and maternal clinical data were available (39). All recordings refer to singleton pregnancies, with more than 36 completed weeks. The CTU-UHB recordings start no more than 90 min before the delivery, and each is at most 90 min long, sampled at 4 Hz (39).

Before labor, the arterial pH of a healthy fetus is around 7.35, whereas, at birth, the average pH of the umbilical artery blood is around 7.25. In this sense, moderate neonatal acidosis will occur when the pH is at least below 7.15 (40). However, not all fetuses in this situation are at risk for immediate or long-term complications, which will depend on the type of acidosis and its severity. Respiratory acidosis *per se* does not carry long-term neurological complications, while metabolic acidosis is related to prolonged hypoxia and needs more time to reverse, even after hypoxia is corrected. By its turn, prolonged metabolic acidosis, involving lower pH values, is associated with irreversible organ damage (40). Fetal metabolic acidosis is defined by an umbilical artery pH below 7.00 and a base-deficit in the extracellular fluid (BD_ecf_) above 12 mmol/l (24, 40, 41). However, there is already an association with adverse short-term perinatal outcomes when pH values are below 7.05 and the BD_ecf_ is above 10 mmol/l (40). Lower arterial pH values are not common in regular cross-sectional studies or even in large randomized controlled trials conducted in the intrapartum setting and considering fetal acidemia as a main outcome measure (42, 43), being a rare event in nowadays' clinical practice (incidence lower than 0.6%) (42), in developed countries. Thus, cutoffs such as 7.20 (31), 7.15 (28), 7.10 (31), and 7.05 (35, 44) are regularly employed in studies of fetal acidemia/hypoxia detection. Accordingly, in this study, four different arterial pH cutoffs were considered for the definition of fetal acidemia: 7.05, 7.10, 7.15, and 7.20. The 7.00 pH cutoff was not considered for this analysis since only three of the included CTGs referred to newborns with an arterial pH below 7.00 (incidence of 0.54%, in line with the aforementioned literature).

#### Signal Pre-processing

Pre-processing of the FHR signal was required to remove artifacts and signal loss that is commonly present in the final minutes before delivery, related to the movements of the descending fetus and the pregnant woman who is pushing and the improper placement of the sensors. The algorithm used, described in detail in (3), detects samples lower than 60 bpm and above 200 bpm and consecutive differences higher than 25 bpm. The segments detected were then replaced by linear interpolation if referring to <2 s. For longer periods, segments were substituted by the previous segment of the same length. All samples were rounded to units. After discarding the last 5 min of each recording, the final 30 min were selected for the following analyses. After pre-processing the available signal, FHR recordings with signal loss above 15% in the last hour (excluding the last 5 min) were rejected, ending with 246 traces selected for spectral analysis.

#### Spectral Analysis

Parseval's theorem states that the total energy of a signal in the time domain is equal to its energy in the frequency domain and, for this reason, the square of the magnitude of the Fourier Transform of a signal represents its power density (22). Using the Fast Fourier Transform (FFT) and the frequency domain bands reported in the literature, various indices of FHR's power spectral density (PSD) were computed.

To estimate the PSD of the FHR, a non-parametric method was employed, the Welch method, with a window length of 256 samples with a 62.5% overlap, similarly to previous studies (3, 11, 13, 31, 34). The Welch periodogram-based estimation method corresponds to integrating the periodogram (using the rectangular method) in windows of overlapped segments, modified periodograms, that are averaged to obtain the PSD estimate, allowing an estimation of the average power of the signal. The integration was done between limited frequency ranges. The FHR power percentages were computed, following previous recommendations (45), corresponding to the power of each frequency band divided by the average power of the whole signal (46). These normalized values allow the detection of relative changes, instead of changes in the total power, that would mask the first ones (25).

Computing a PSD estimation directly on the FHR signal results in a high impulse around 0 Hz frequency, the DC component, corresponding to the signal's average value over a period, disguising relevant signals with relatively small amplitude. Thus, after preprocessing the signals, and before the spectrum estimation, the mean FHR was removed for each signal to reduce the DC-offset of FHR. All preprocessing computations and spectral analysis were done using MATLAB (R2020b, MathWorks, Natick, MA, USA).

### Statistical Analysis

Descriptive measures and statistical tests were employed to properly compare the FHR power percentage of the acidemic and the non-acidemic fetuses on various frequency bands. The normality of quantitative variables was verified visually and confirmed with the Kolmogorov-Smirnov test. Since almost all indices could not be assumed as normally distributed, median and interquartile interval (first quartile, Q1 - third quartile, Q3) were employed. Categorical variables were described by absolute and relative frequencies.

For the comparison between acidemic and non-acidemic groups of fetuses, the Mann-Whitney test was employed, comparing the distribution functions of the spectral indices between the two groups. Subsequently, and for the frequency bands where significant differences were found, a receiver operating characteristic (ROC) curve was computed (47). The spectral indices' ability to distinguish acidemic fetuses from non-acidemic ones was evaluated using the area under the ROC curve (AUROC). These curves relate sensitivity—ability to recognize acidemic fetuses correctly—and specificity—ability to identify non-acidemic fetuses—for each cutoff of the discriminating index (31), or power spectral band, in our case.

For descriptive and inference statistics, SPSS Statistics (v.25; IBM SPSS, Chicago, IL) was used. For all statistical tests, a significance level of 0.05 was pre-defined.

## Results

### Literature Review of Spectral Analysis of FHR

Overall, a total of 176 papers were reviewed by abstract. At the abstract screening step, 73 papers were excluded for several reasons: 6 referring to animal studies, 6 to reviews (with no signal analyses), 14 did not analyze FHR nor RR, 33 did not apply power spectral analysis at all, and finally, 14 were excluded as they used magnetocardiography for FHR acquisition. As for the analyses of full-text articles for eligibility, another 76 documents were excluded for the same set of reasons described in the previous step (Figure 1) together with: one article which referred to the analysis of simulated signal and 48 studies reporting analysis of antepartum signals (plus two with no information). Three additional articles obtained from reference checking were also included. In the end, 26 different original papers reporting spectral analysis of intrapartum FHR (or RR intervals) were included in the analysis, from 1975 to 2019.

The most frequent aim in the studies included was the assessment of acidemia state alone (*n* = 14) or combined with other characteristics of the fetus such as gender (32) or prematurity (48). There was also a study addressing the relation between PSD indices and cord arterial base deficit values at birth (49). Other than acidemia, the study of the impact of signal preprocessing for artifact correction on PSD features (50), and the effect of different acquisition modes (3), were the aim of other studies. Power spectral analysis was also employed for the characterization/detection of a great diversity of other situations: maturity (19, 20), weight or/and gender (21, 22), and conditions of fetal distress (23). Included articles described analysis on FHR and RR-intervals, mainly acquired through fetal scalp ECG (*n* = 14) and fetal Doppler transducer (external ultrasound, *n* = 12). The majority of articles (*n* = 14) reported the use of the Fast Fourier Transform method for computing the spectral density of the signals, without further details. From the remaining studies, six employed the Welch method, three the autoregressive model, one used the matching pursuit technique (51), and the other used the standard periodogram method (35). Regarding signal and sample rate, the great majority of the articles included analyzed FHR (*n* = 22) signals sampled at 4 Hz (*n* = 10) or 2 Hz (*n* = 5). Other sampling rates were also analyzed, although less frequently, such as 1, 10, and 16 Hz. From the 4 studies reporting power spectral analysis on RR signals, three used signals sampled at 4 Hz.

To address the main aim of this study, all the frequency bands used in the power spectral analysis were extracted from the 26 articles included in the revision. A wide variety of frequency bands' limits was found, and only two articles did not report the use of any specific interval or frequency bands: one from 1975 aiming at characterizing spectral density for premature infants or prolonged labor (19) and a more recent one aiming at computing FHR from beat-to-beat signal (52). From the remaining 24 (Table 1), regarding very low frequency, the bands' limits employed were 0–0.03 Hz in seven articles, 0–0.04 Hz in three, and 0.003–0.004 Hz in one study. In contrast, thirteen studies did not refer to the very low frequency power energy. From the eleven studies reporting the power in the very low frequency band, six attributed its activity to the thermoregulation and slow regulating systems of peripheral vessels, as stated in 1996 Task Force (29). One study reported the use of an intermediate frequency, named as low low frequency (LLF), referring to the range 0.04–0.08 Hz (51).

**Table 1:** Information on different frequency bands and related contents of the 24 articles reviewed.

**Frequency band (Hz)**	**Signal-sampling rate**	**PSD method**	**Main goal**	**Origin of the bands (and their references)**	**Publication year (article reference)**
VLF (0–0.04)LF (0.04–0.15)HF (0.15–0.4)VHF (0.75–1.5)	FHR – 2 Hz	FFT	Diagnose fetal acidemia	Adults	2001 (28)
LF (0.03–0.07)MF (0.07–0.13)HF (0.13–1)	FHR – 4 Hz	FFT	Association PSD with fetal cord arterial base deficit values at birth	Adults (53–55)	2001 (49)
LF (0.04–0.15)HF (0.15–1.0)	FHR – 16 Hz	FFT	Diagnose fetal acidemia	Adults	2005 (44)
	RR – 500 Hz				2007 (56)
	FHR – 16Hz				2013 (57)
LF (0.04–0.15)HF (0.15–0.4)	FHR – 10 Hz	Welch method	Diagnose fetal acidemia	Adults	2015 (11)
VLF (0.003–0.04)LF (0.04–0.15)HF (0.15–0.4)	FHR – 10 Hz	Welch method	Diagnose fetal acidemia	Adults	2017 (13)
VLF (0–0.04)LF (0.04–0.15)HF_A_ (0.15–0.4)HF_N_ (0.4–1.5)	RR – 4 Hz	FFT	Compare real signal with artifacts and without artifacts	Neonates and Adults	2008 (50)
LF (0.04–0.15)HF (0.4–1.5)	FHR – 4 Hz	FFT	Compare near to post term during active and quiet sleep	Neonates (45, 58)	2009 (20)
	RR – 4 Hz	FFT	Diagnose fetal acidemia		2010 (27)
		FFT			2011 (59)
VLF (0–0.03)LF (0.03–0.15)MF (0.15–0.5)HF (0.5–1)	FHR – 2 Hz	Welch method	Diagnose fetal acidemia	Fetuses (Antepartum) (17)	2006 (31)
	FHR – 2 and 4 Hz	Welch method	Internal vs. external acquisition modes		2006 (3)
	FHR – 4 Hz	Not reported	Diagnose Fetal Acidemia/gender differences		2009 (32)
		Autoregressive model	Diagnose fetal acidemia		2011 (33)
		Welch method	Indices in beat-to-beat vs. 4 Hz		2013 (34)
		Welch method	Differences in fetal gender		2017 (60)
		Periodogram	Diagnose fetal acidemia		2019 (35)
LF (0.03–0.15)MF (0.15–0.5)HF (0.5–1)	FHR – 4 Hz	Autoregressive model	Diagnose fetal acidemia	Fetuses (Antepartum) (17)	2010 (61)
LF (0.04–0.15)MF (0.15–0.5)HF (0.5–1)	FHR – 2 Hz	FFT	Diagnose fetal acidemia and prematurity	Fetuses (Antepartum) (17)	2012 (48)
			Differences in fetal weight and gender		2014 (21)
VLF (0–0.04)LLF (0.04–0.08)LF (0.08–0.15)HF (>0.15)	FHR – 1 Hz	Matching pursuit	Diagnose fetal acidemia	Not mentioned	2006 (51)
LF (0.03125–0.1)	FHR – 2 Hz	FFT	Diagnose fetal distress	Not mentioned	2010 (23)
LF (0.02–0.14)MF (0.1–0.4)HF (0.4–1.4)	FHR – 4 Hz	Autoregressive model	Diagnose fetal acidemia	Not mentioned	2015 (62)

*VLF, very low frequency; LLF, low low frequency; LF, low frequency; MF, movement frequency; HF, high frequency; VHF, very high frequency; FHR, fetal heart rate; RR, interval between R peaks; PSD, power spectral density; FFT, fast Forrier transform*.

The low frequency was addressed in all the 24 articles, where the most common band was within the interval 0.04–0.15 Hz, used in 12 studies, followed by 0.03–0.15 Hz, addressed by 8 articles. Regarding the power in the LF band, most articles attribute it to the activity of the sympathetic system (3, 28, 31, 32, 34, 49, 59, 60, 62), while others link it to the combined activity of sympathetic and parasympathetic activity (11, 13, 20, 21, 44, 48, 57, 61). This different interpretation cannot, in some cases at least, be attributed to the different frequency limits of the bands used for the LF computation (check Table 1), and is probably inherited by the same controversial interpretation of the LF component in adults' heart rate analysis (29).

Only 12 studies assessed the power in the movement frequency, and ten of them computed its energy within the limits 0.15–0.5 Hz. One article reported limits of 0.07–0.13 Hz for the MF band (49). Fetal movements and maternal breathing are the activities most associated to the MF band (3, 21, 34, 62), with only one article (49) attributing both sympathetic and parasympathetic nervous control to the activity in this frequency band. This difference in the interpretation can be attributed to the different ranges in the MF band, which shows a large superposition with the LF component.

The high frequency band was employed in 23 studies, and the interval 0.5–1 Hz was the most used, being reported in 10 articles. Only one study mentioned the use of a very high frequency band, within 0.75–1.5 Hz, aiming to the detection of fetal acidemia (28). The majority of the articles addressing the HF band interpreted its power as reflecting the activity on the parasympathetic systems, as advocated by the 1996 Task Force (29), and only a few specify the connection to fetal breathing (61, 62) due to the vagal nerve (28). The VHF was reported once, interpreted as reflecting the domain of the heart beating (28).

Regarding the ratio of spectral bands, interpreted as the sympathovagal balance in the control of heart rate activity (between the parasympathetic and the sympathetic branches), three of the included articles reported the use of LF/(MF + HF) and eight, who did not report the use of movement frequency band [with one exception (21)], preferred LF/HF. Three studies reported both ratio measures LF/(MF + HF) and LF/HF (32, 34, 48), referring that the two ratios provide different balances between the autonomic nervous system branches.

No clear relation was found between the type of signal analyzed or sampling rate regarding the bands adopted.

From the 24 papers using specific frequency bands, seven refer to bands inspired in human adults' signals, four in newborns and ten used bands defined based on power spectral analysis of the FHR, spanning a wide range of publication years (2001–2017 for adult bands and 2006–2019 for fetal analysis inspired bands) (Table 1).

### Spectral Analysis on CTG Intrapartum Open-Access Database

**Figure 2:**
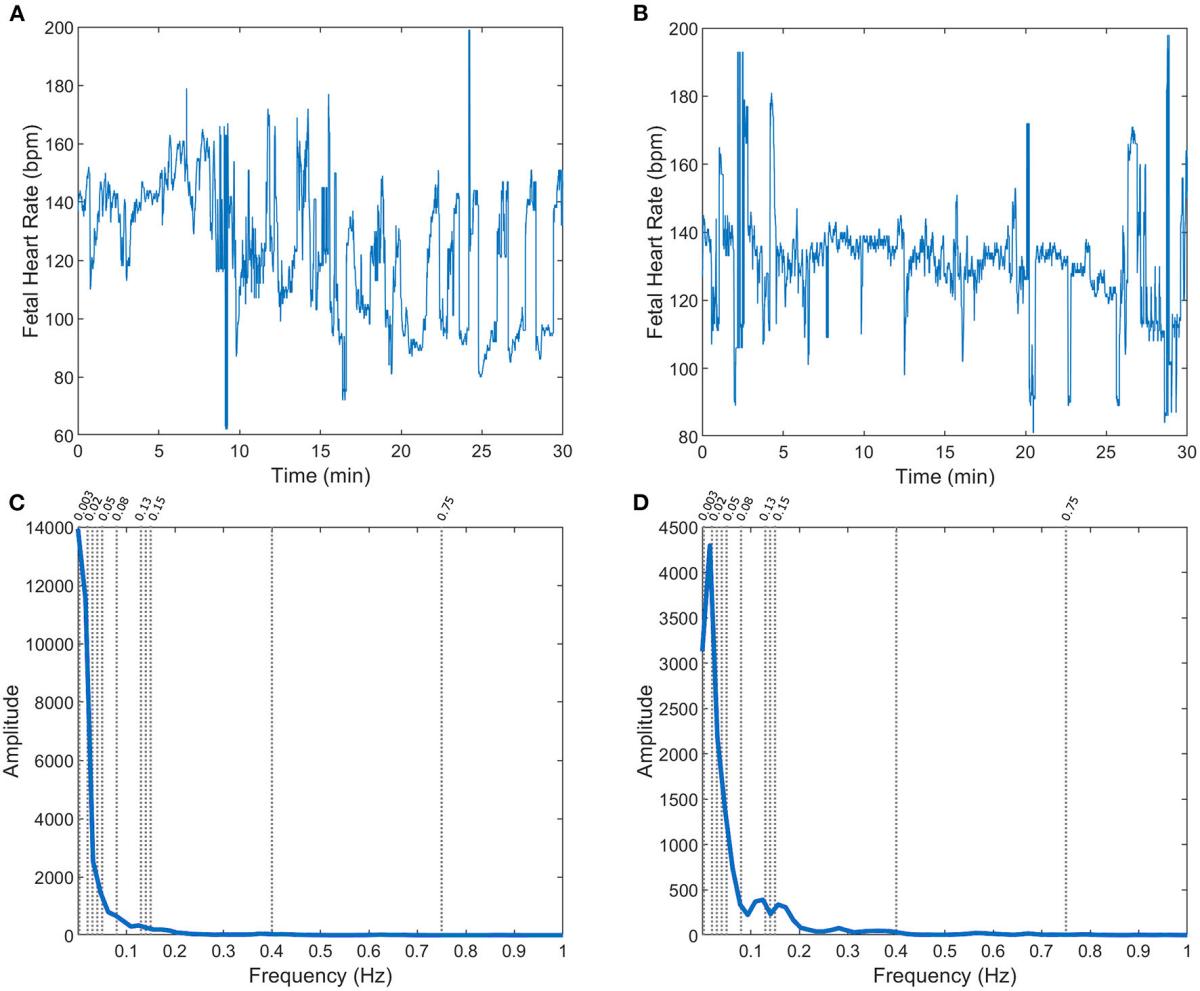
FHR **(A,B)** and respective power spectra (**C,D**, with specific frequency limits) for two fetuses in the CTG database: with arterial blood pH = 7.02 **(A,C)** and pH = 7.2 **(B,D)**, at birth.

A total of 246 FHR recordings were included in the analysis, referring to the last half an hour (excluding the last 5 min) of the recordings from the CTU-UHB open-access database, with <15% of signal loss. The FHR signals were slightly more often from female fetuses (51%), with occipital presentation (91%), a median (Q1–Q3) of 40 (39–41) weeks of gestation, and mother's age of 29 (26.8–32) years old. Only 7, 12, 39, and 72 cases were classified as acidemic babies, considering arterial blood pH cutoffs of 7.05, 7.10, 7.15, and 7.20, respectively (Figure 2 - two FHR examples with the respective power spectral estimate).

Descriptive statistics of the several spectral indices computed on the 246 CTU-UHB recordings are presented in Supplementary Tables 1–4, together with their comparison between acidemic and non-acidemic groups of fetuses. Acidemic groups were defined for each pH cutoff, namely pH ≤ 7.20, pH ≤ 7.15, pH ≤ 7.10, and pH ≤ 7.05. Overall, acidemic cases pointed to higher values of VLF power and lower powers in all other spectral bands (LF, LLF, MF, HF, and VHF ranges), and significant differences were found between the two groups defined by all four pH cutoffs, for some spectral bands (Supplementary Tables 1–3). In particular, for cutoff 7.20, significant differences were found only for VHF band 0.75–1.5 Hz (28) and a low AUROC of 0.593 was obtained (see Table 2). For cutoff 7.15, significant differences between acidemic and non-acidemic groups were detected for VLF, LLF, LF, and VHF indices (Supplementary Table 2), and their AUROC were computed (Table 3). For those, the best AUROC values were: 0.626 for LF 0.04–0.15 Hz (11, 13, 20, 21, 27, 28, 44, 48, 50, 56, 57, 59) and 0.624 for LLF 0.04–0.08 Hz (51) and for LF 0.03125–0.1 Hz (23), as depicted in Table 3.

**Table 2:** Area under ROC curve and correspondent non-parametric confidence interval, for the bands significantly different between groups of fetal acidemia defined with pH cutoff of 7.20, in the CTU-UHB open-access database (section Spectral Analysis on CTG Intrapartum Open-Access Database).

**Frequency band (Hz) (reference)**	**Area**	**95% confidence interval**
**VHF (0.75–1.5)** (28)	0.593	(0.514–0.672)

*VHF, very high frequency*.

**Table 3:** Areas under ROC curves and correspondent non-parametric confidence interval, for the band significantly different between groups of fetal acidemia defined with pH cutoff of 7.15, in the CTU-UHB open-access database (section Spectral Analysis on CTG Intrapartum Open-Access Database).

**Frequency band (Hz)** **(references)**	**Area**	**95% confidence interval**
**VLF (0–0.03)** (3, 31–35, 60)	0.610	(0.521–0.699)
**LLF (0.04–0.08)** (51)	**0.624**	**(0.533–0.716)**
**LF (0.02–0.14)** (62)	0.617	(0.520–0.714)
**LF (0.03–0.07)** (49)	0.617	(0.521–0.713)
**LF (0.03–0.15)** (3, 31–35, 60, 61)	0.617	(0.521–0.714)
**LF (0.03125–0.1)** (23)	**0.624**	**(0.532–0.715)**
**LF (0.04–0.15)** (11, 13, 20, 21, 26–28, 44, 48, 50, 56, 57, 59)	**0.626**	**(0.534–0.717)**
**VHF (0.75–1.5)** (28)	0.615	(0.519–0.710)

*In bold are the three best AUROC values. VLF, very low frequency; LLF, low low frequency; LF, low frequency; VHF, very high frequency*.

For cutoff 7.10, significant differences were found for all spectral bands' indices (Supplementary Table 3), and highest AUROC values were achieved, comparing with the previous acidemic definition (see Table 4). Overall, the best AUROC values were: 0.731 for LF 0.04–0.15 Hz (11, 13, 20, 21, 27, 28, 44, 48, 50, 56, 57, 59); 0.730 for LF 0.03125–0.1 Hz (23) and 0.729 for LLF 0.04–0.08 Hz (51) (see Table 4).

**Table 4:** Areas under ROC curves and correspondent non-parametric confidence interval, for the bands significantly different between groups of fetal acidemia defined with pH cutoff of 7.10, in the CTU-UHB open-access database (section Spectral Analysis on CTG Intrapartum Open-Access Database).

**Frequency band (Hz)(references)**	**Area**	**95% confidence interval**
**VLF (0–0.03)** (3, 31–35, 60)	0.724	(0.597–0.852)
**VLF (0–0.04)** (28, 50, 51)	0.717	(0.584–0.851)
**VLF (0.003–0.04)** (13)	0.717	(0.584–0.851)
**LLF (0.04–0.08)** (51)	**0.729**	**(0.598–0.860)**
**LF (0.02-0.14)** (62)	0.703	(0.544–0.863)
**LF (0.03–0.07)** (49)	0.700	(0.542–0.859)
**LF (0.03–0.15)** (3, 31–35, 60, 61)	0.703	(0.543–0.864)
**LF (0.03125-0.1)** (23)	**0.730**	**(0.601–0.859)**
**LF (0.04–0.15)** (11, 13, 20, 21, 26–28, 44, 48, 50, 56, 57, 59)	**0.731**	**(0.605–0.856)**
**LF (0.08−0.15)** (51)	0.710	(0.583–0.837)
**MF (0.07−0.13)** (49)	0.722	(0.597–0.846)
**MF (0.1-0.4)** (62)	0.698	(0.564–0.832)
**MF (0.15–0.5)** (3, 21, 31–35, 48, 60, 61)	0.680	(0.542–0.818)
**HF (>0.15)** (51)	0.684	(0.538–0.829)
**HF (0.13-1)** (49)	0.677	(0.533–0.821)
**HF (0.15–0.4)** (11, 13, 28)	0.683	(0.548–0.818)
**HF (0.15–1.0)** (44, 56, 57)	0.679	(0.535–0.824)
**HF (0.4–1.5)** (20, 27, 50, 59)	0.678	(0.512–0.844)
**HF (0.4–1.4)** (62)	0.675	(0.509–0.841)
**HF (0.5–1)** (3, 21, 31–35, 48, 61)	0.673	(0.511–0.835)
**VHF (0.75–1.5)** (28)	0.702	(0.540–0.865)

*In bold are the three best AUROC values. VLF, very low frequency; LLF, low low frequency; LF, low frequency; MF, movement frequency; HF, high frequency; VHF, very high frequency*.

For cutoff 7.05, although the acidemic group had only 7 subjects, significant differences were found for VLF and LF spectral bands (Supplementary Table 4), and their AUROC were computed (Table 5). Overall, the best AUROC values were: 0.770 for LF 0.03–0.07 Hz (49); 0.763 for LF 0.02–0.14 Hz (62) and 0.762 for LF 0.03–0.15 Hz (3, 31–35, 60) (see Table 5).

**Table 5:** Areas under ROC curves and correspondent non-parametric confidence interval, for the bands significantly different between groups of fetal acidemia defined with pH cutoff of 7.05, in the CTU-UHB open-access database (section Spectral Analysis on CTG Intrapartum Open-Access Database).

**Frequency band (Hz)(references)**	**Area**	**95% confidence interval**
**VLF (0–0.03)** (3, 31–35)	0.692	(0.514–0.870)
**LLF (0.04–0.08)** (51)	0.759	(0.617–0.900)
**LF (0.02-0.14)** (62)	**0.763**	**(0.593–0.932)**
**LF (0.03–0.07)** (49)	**0.770**	**(0.608–0.932)**
**LF (0.03–0.15)** (3, 31–35, 60, 61)	**0.762**	**(0.589–0.936)**
**LF (0.03125-0.1)** (23)	0.759	(0.616–0.902)
**LF (0.04–0.15)** (11, 13, 20, 21, 26–28, 44, 48, 50, 56, 57, 59)	0.759	(0.611–0.906)

*In bold are the three best AUROC values. VLF, very low frequency; LLF, low low frequency; LF, low frequency*.

## Discussion

A total of 26 articles were included in the systematic review presented here, surveying for spectral analysis of intrapartum fetal heart rate. As suspected, a great panoply of frequency bands has been applied, some inspired in fetal heart rate spectrum evidence and others in adult and neonatal heart rate studies. Although it seems, from our results, that most recent studies preferably select frequency bands inspired in the fetus, as normally expected, we also found some recent studies choosing a spectrum of adult-derived bands. This finding might reflect that there is some controversy regarding the proper bands to use in fetuses (11, 13). In a study comparing the performance of spectral analysis and the Hurst parameter for fetal acidemia detection, the Hurst parameter revealed to be a potential marker of fetal acidosis, overcoming the performance of the spectral index LF/HF, with the advantage of not depending on the choice of the partitioning of frequency bands (11). This interest in providing an alternative to the spectrum splitting is also fostered by the fact that several conditions impact the association between the power in the spectral bands and ANS activity in the fetus, such as maturity differences between the sympathetic and parasympathetic systems; uterine contractions affecting the intrathoracic fetal pressure, which influence the FHR absolute values and variability (63, 64). As noticed in a 2008 review by Van Laar et al. (45), the different heart rate and pattern of breathing movements of fetuses compared to adults suggest that adult-inspired frequency bands may not be perfectly chosen for FHR analysis, and their recommendation of an agreement on the frequency bands chosen remains present. Nevertheless, several studies have been conducted for fetal acidosis detection, wherefrom all the bands and ratios used, the VLF and LF bands are revealed as promising in separating acidemic groups (13, 27, 33, 35, 51, 59).

The early detection of acidemic fetuses was the most common aim (16/26) in the papers included in the systematic review. The capacity of each individual spectral band found in the literature, computed on the 246 FHR's signals for discriminating acidemic from non-acidemic fetuses was accessed, for four pH cutoffs: 7.05; 7.10; 7.15 and 7.20. The highest AUC values were obtained when using lower pH cutoffs. Lower cutoffs are indicative of more severe acidemia, which probably causes a more notorious change in the variability of the fetal heart rate. This allows a more efficient detection of acidemic fetuses, in particular using power spectral analysis, although it is commonly hampered by the small number of severely acidemic fetuses in available datasets (42, 43). Considering the detection of acidemic fetuses with pH ≤ 7.05, the best frequency bands, with AUC above 0.760, were all low frequency bands. This indicates that the low frequency band might be the best at distinguishing acidemic from non-acidemic fetuses, at least in more severe cases.

The LF bands with the highest AUC, for a cutoff of 7.05, have their origins in different studies, from fetuses, 0.03–0.15 Hz (17), 0.02–0.14 Hz (62), to adults-based, 0.03–0.07 Hz (49). Differences between these frequency ranges are small on the lower limits (0.02–0.03) but include, in the upper limits, larger differences (from 0.07 to 0.15) (17, 49). Results for this severe group should be considered with caution, due to the fact that this was a very limited set of cases, with only seven fetuses. The main limitation of this study relates to the very low incidence of severe acidemic fetuses in this FHR database, which is in accordance with previously published studies (42, 43). This limitation, combined with many band cutoffs used, has resulted in a very wide CI of the AUC. This methodology needs to be validated in a larger dataset (combination of clinical datasets) with an increased prevalence of acidemic fetuses to overcome this limitation.

For the less severe cutoffs, 7.10 and 7.15, results were similar, with the same three low frequency bands as the most promising: 0.04–0.08 (51); 0.03125–0.1 (23) and 0.04–0.15 (11, 13, 20, 21, 26–28, 44, 48, 50, 56, 57, 59). For these, the last was inspired in evidence stemming from neonatal/adult spectra (29, 45, 58), while the two previous were probably derived from empirical studies. These results do not confirm our intuition that the bands derived from FHR spectra should be more accurate for the detection of pathological cases. Indeed, the human fetus has a different heart rate and distinct pattern of breathing movements compared to a human adult or even newborns (45). Additionally, in all discriminative LF bands presented, the reduced power found in the case of acidemic cases was in accordance with previous studies (45).

Several studies (18/24) do not evaluate the VLF bands. This approach might be related to the attempt to quantify changes in the FHR baseline. To our views, clinically, in the case of sub-acute fetal hypoxia (slowly evolving hypoxia, e.g., as a result of non-reversed excessive uterine contractions during labor), the change of variability in the baseline, especially after an increased baseline shift, is very informative and therefore is not essential to evaluate LF or VLF bands. On the other hand, in acute events, such as a cord prolapse, a decrease of variability within the deceleration is specifically related to fetal hypoxia (2), and therefore the evaluation of the LF or VLF bands must be considered.

In this review, we have chosen to restrict our analysis to the intrapartum period, in an attempt to provide homogeneity to the included studies, reducing the variation of the encountered frequency bands. There is evidence that the spectral density of spectral bands is changed not only by the resting state of the fetus (38), but also, and easier to control, by the gestational age or developmental maturity (64).

Despite the multitude of definitions encountered for frequency bands, methods used and signals employed in the conducted literature review, the consistency of the results across three of the four definitions of fetal acidemia here evaluated and the performance metrics' values obtained, spectral analysis remains a powerful method to understand the dynamics of the fetal autonomic system. Studies encompassing more detailed characterization of the included subjects, namely the fetus' physiological state, should be fostered in order to develop a better performance of spectral based indices and to get them ready to be incorporated in computerized systems that aid in the clinical assessment of fetal well-being. Regarding the power spectrum of antepartum signals, and given the difficulty in providing a standard recommendation for the selection of spectral bands, the idea of partitioning the power spectrum in empirical bands, or in consecutive bands of equal bandwidth, might be a secure approach (52, 64, 65) in comparison to the compromise of choosing predefined band definitions.

Behind the efforts reflected in many studies to obtain an effective computational tool to disclose fetal distress, is the hypothesis that the frequency decomposition of the FHR can help clinicians to predict it, specifically metabolic acidosis. This could be an adjunctive methodology to the CTG that would help clinicians to implement adequate obstetrical interventions, before metabolic acidosis is severe enough to produce irreversible damages.

## Conclusion

In conclusion, this work shows that, in general, most research papers do not agree on the definition of frequency bands to be used, which is essential for their application in clinical practice. The low frequency bands are the most promising in detecting fetuses at risk of acidosis. Furthermore, the power spectrum analysis of FHR is a powerful tool to help physicians diagnose fetal acidemia in the intrapartum period. Thus, it is essential to determine an exact diagnostic value of spectral analysis to be considered for the identification of fetuses at risk of severe acidosis and to standardize the methods used in signal pre-processing and in the spectral analysis itself to allow a better comparison between studies. In addition, to enable adequate clinical applicability, a focus on real-time intrapartum fetal monitoring should also be a priority in future research.

## Data Availability Statement

The raw data supporting the conclusions of this article will be made available by the authors, without undue reservation.

## Author Contributions

LC, TH, and IN: conceptualization and methodology. ML, LC, and TH: formal analysis and investigation. ML and LC: writing—original draft preparation. ML, LC, TH, and IN: writing—review and editing. All authors have read and agreed to the published version of the manuscript.

## Conflict of Interest

The authors declare that the research was conducted in the absence of any commercial or financial relationships that could be construed as a potential conflict of interest.

## Publisher's Note

All claims expressed in this article are solely those of the authors and do not necessarily represent those of their affiliated organizations, or those of the publisher, the editors and the reviewers. Any product that may be evaluated in this article, or claim that may be made by its manufacturer, is not guaranteed or endorsed by the publisher.

## Abbreviations

AUROC: area under the receiver operating characteristic curve
CTG: cardiotocography
FHR: fetal heart rate
HF: high frequency
LF: low frequency
LLF: low low frequency
MF: movement frequency
PSD: power spectral density
ROC: receiver operating characteristic
VLF: very low frequency.
