# Supplementary material for: Systematic Review of Intrapartum Fetal Heart Rate Spectral Analysis and an Application in the Detection of Fetal Acidemia
Source: Front Pediatr. 2021 Aug 2;9:661400. doi: 10.3389/fped.2021.661400 (PMC8364976; doi:10.3389/fped.2021.661400)
Supplement: Supplementary file 1 [file Data_Sheet_1.PDF]

Supplementary Material

Table S1 - Comparison of spectral bands energy between non-acidemic (n=174) and acidemic fetuses (n=72), for a pH cutoff of 7.20

Frequency Band (Hz)	References	Total	Non-acidemic	Acidemic	Mann-Whitney p-value
**VLF (0 - 0.03), med (Q1-Q3)**	(3, 31-35, 53)	84.25 (79.19 - 89.82)	84.18 (78.89 - 89.18)	84.26 (80.62 - 91.47)	0.173
**VLF (0 - 0.04), med (Q1-Q3)**	(28, 48, 49)	88.59 (84.67 - 92.6)	88.82 (84.27 - 92.08)	88.05 (85.48 - 93.71)	0.347
**VLF (0.003 - 0.04), med (Q1-Q3)**	(13)	88.59 (84.67 - 92.6)	88.82 (84.27 - 92.08)	88.05 (85.48 - 93.71)	0.347
**LLF (0.04 – 0.08), med (Q1-Q3)**	(49)	17.17 (12.37 - 22.85)	17.97 (12.91 - 23.16)	16.36 (10.15 - 22.01)	0.056
**LF (0.02-0.14) med (Q1-Q3)**	(51)	53.33 (47.49 - 60.48)	53.85 (48.44 - 60.48)	52.7 (45.05 - 60.32)	0.083
**LF (0.03 - 0.07) med (Q1-Q3)**	(47)	49.85 (43.98 - 55.51)	50.35 (44.78 - 55.87)	49.04 (42.13 - 54.7)	0.072
**LF (0.03 - 0.15), med (Q1-Q3)**	(3, 31-35, 53, 55)	53.76 (47.87 - 60.86)	54.26 (48.7 - 60.97)	53.22 (45.32 - 60.78)	0.092
**LF (0.03125-0.1) med (Q1-Q3)**	(23)	18.38 (13.18 - 23.94)	18.97 (13.51 - 24.05)	17.21 (10.69 - 22.88)	0.062
**LF (0.04 - 0.15), med (Q1-Q3)**	(11, 13, 20, 21, 27, 28, 42, 46, 48, 52, 54, 59, 61)	20.14 (14.3 - 26.54)	20.87 (14.58 - 26.58)	19.29 (11.74 - 24.93)	0.066
**LF (0.08 -0.15), med (Q1-Q3)**	(49)	5.46 (3.55 - 7.82)	5.4 (3.94 - 7.98)	5.68 (3.26 - 7.4)	0.364
**MF (0.07 -0.13), med (Q1-Q3)**	(47)	7.54 (5.19 - 10.75)	7.47 (5.49 - 10.85)	7.88 (4.15 - 10.47)	0.323

**MF (0.1-0.4), med (Q1-Q3)**	(51)	6.2 (3.96 - 8.25)	6.1 (4.21 - 8.33)	6.32 (3.32 - 7.88)	0.410
**MF (0.15 - 0.5), med (Q1-Q3)**	(3, 21, 31-35, 46, 53, 55)	3.5 (2.15 - 4.6)	3.47 (2.29 - 4.63)	3.59 (1.91 - 4.51)	0.359
**HF (>0.15), med (Q1-Q3)**	(49)	4.15 (2.61 - 5.53)	4.12 (2.78 - 5.63)	4.18 (2.32 - 5.35)	0.277
**HF (0.13-1), med (Q1-Q3)**	(47)	4.74 (3.02 - 6.21)	4.64 (3.2 - 6.32)	4.8 (2.64 - 6.1)	0.333
**HF (0.15 - 0.4), med (Q1-Q3)**	(11, 13, 28)	3.32 (2.03 - 4.33)	3.25 (2.18 - 4.42)	3.38 (1.78 - 4.27)	0.362
**HF (0.15 - 1.0), med (Q1-Q3)**	(42, 54, 59)	3.96 (2.49 - 5.25)	3.93 (2.66 - 5.34)	3.98 (2.19 - 5.07)	0.310
**HF (0.4 - 1.5), med (Q1-Q3)**	(20, 27, 48, 52)	0.95 (0.58 - 1.34)	0.96 (0.61 - 1.42)	0.84 (0.49 - 1.25)	0.101
**HF (0.4 - 1.4), med (Q1-Q3)**	(51)	0.92 (0.56 - 1.3)	0.93 (0.59 - 1.37)	0.81 (0.47 - 1.21)	0.105
**HF (0.5 - 1), med (Q1-Q3)**	(3, 21, 31-35, 46, 55)	0.47 (0.28 - 0.68)	0.48 (0.3 - 0.69)	0.42 (0.24 - 0.62)	0.099
**VHF (0.75 - 1.5), med (Q1-Q3)**	(28)	0.38 (0.23 - 0.51)	0.39 (0.24 - 0.54)	0.31 (0.2 - 0.47)	**0.022**

Bold: significant p-values. Abbreviations: VLF, very low frequency; LLF, low low frequency; LF, low frequency; MF, movement frequency; HF, high frequency; VHF, very high frequency; med, median; Q1, first quartile; Q3, third quartile.

Table S2 - Comparison of spectral bands energy between non-acidemic (n=207) and acidemic fetuses (n=39), for a pH cutoff of 7.15

Frequency Band (Hz)	References	Total	Non-acidemic	Acidemic	Mann-Whitney p-value
**VLF (0 - 0.03), med (Q1-Q3)**	(3, 31-35, 53)	84.25 (79.19 - 89.82)	83.85 (78.82 - 89.71)	86.45 (82.47 - 91.3)	**0.029**
**VLF (0 - 0.04), med (Q1-Q3)**	(28, 48, 49)	88.59 (84.67 - 92.6)	88.48 (84.06 - 92.56)	90.58 (86.28 - 93.79)	0.053

**VLF (0.003 - 0.04), med (Q1-Q3)**	(13)	88.59 (84.67 - 92.6)	88.48 (84.06 - 92.56)	90.58 (86.28 - 93.79)	0.053
**LLF (0.04 – 0.08), med (Q1-Q3)**	(49)	17.17 (12.37 - 22.85)	18.16 (12.84 - 23.16)	15.76 (9.83 - 18.39)	**0.014**
**LF (0.02-0.14) med (Q1-Q3)**	(51)	53.33 (47.49 - 60.48)	53.89 (47.79 - 60.71)	50.96 (42.66 - 57.7)	**0.020**
**LF (0.03 - 0.07) med (Q1-Q3)**	(47)	49.85 (43.98 - 55.51)	50.36 (44.45 - 55.93)	47.19 (40.95 - 51.22)	**0.021**
**LF (0.03 - 0.15), med (Q1-Q3)**	(3, 31-35, 53, 55)	53.76 (47.87 - 60.86)	54.28 (48.02 - 61.29)	51.26 (42.78 - 58.02)	**0.020**
**LF (0.03125-0.1) med (Q1-Q3)**	(23)	18.38 (13.18 - 23.94)	18.95 (13.4 - 24.15)	16.99 (10.36 - 19.36)	**0.014**
**LF (0.04 - 0.15), med (Q1-Q3)**	(11, 13, 20, 21, 27, 28, 42, 46, 48, 52, 54, 59, 61)	20.14 (14.3 - 26.54)	20.87 (14.58 - 26.59)	18.32 (11.57 - 21.66)	**0.013**
**LF (0.08 -0.15), med (Q1-Q3)**	(49)	5.46 (3.55 - 7.82)	5.55 (3.87 - 7.98)	4.72 (3.2 - 7.25)	0.057
**MF (0.07 -0.13), med (Q1-Q3)**	(47)	7.54 (5.19 - 10.75)	7.87 (5.42 - 11)	6.46 (4.28 - 8.79)	0.062
**MF (0.1-0.4), med (Q1-Q3)**	(51)	6.2 (3.96 - 8.25)	6.24 (4.15 - 8.4)	5.16 (3.28 - 7.45)	0.067
**MF (0.15 - 0.5), med (Q1-Q3)**	(3, 21, 31-35, 46, 53, 55)	3.5 (2.15 - 4.6)	3.57 (2.24 - 4.8)	2.67 (1.91 - 4.33)	0.086
**HF (>0.15), med (Q1-Q3)**	(49)	4.15 (2.61 - 5.53)	4.16 (2.72 - 5.82)	3.06 (2.3 - 5.09)	0.077
**HF (0.13-1), med (Q1-Q3)**	(47)	4.74 (3.02 - 6.21)	4.76 (3.12 - 6.55)	3.46 (2.68 - 5.67)	0.071
**HF (0.15 - 0.4), med (Q1-Q3)**	(11, 13, 28)	3.32 (2.03 - 4.33)	3.4 (2.11 - 4.51)	2.51 (1.77 - 3.98)	0.078
**HF (0.15 - 1.0), med (Q1-Q3)**	(42, 54, 59)	3.96 (2.49 - 5.25)	4 (2.59 - 5.51)	2.97 (2.18 - 4.86)	0.081

**HF (0.4 - 1.5), med (Q1-Q3)**	(20, 27, 48, 52)	0.95 (0.58 - 1.34)	0.96 (0.59 - 1.4)	0.78 (0.49 - 1.2)	0.076
**HF (0.4 - 1.4), med (Q1-Q3)**	(51)	0.92 (0.56 - 1.3)	0.93 (0.57 - 1.34)	0.75 (0.46 - 1.17)	0.083
**HF (0.5 - 1), med (Q1-Q3)**	(3, 21, 31-35, 46, 55)	0.47 (0.28 - 0.68)	0.48 (0.29 - 0.69)	0.41 (0.23 - 0.58)	0.080
**VHF (0.75 - 1.5), med (Q1-Q3)**	(28)	0.38 (0.23 - 0.51)	0.39 (0.24 - 0.54)	0.29 (0.16 - 0.44)	**0.023**

Bold: significant p-values. Abbreviations: VLF, very low frequency; LLF, low low frequency; LF, low frequency; MF, movement frequency; HF, high frequency; VHF, very high frequency; med, median; Q1, first quartile; Q3, third quartile.

Table S3 - Comparison of spectral bands energy between non-acidemic (n=234) and acidemic fetuses (n=12), for a pH cutoff of 7.10

Frequency Band (Hz)	References	Total	Non-acidemic	Acidemic	Mann-Whitney p-value
**VLF (0 - 0.03), med (Q1-Q3)**	(3, 31-35, 53)	84.25 (79.19 - 89.82)	84.11 (78.95 - 89.18)	90.83 (83.22 - 92.24)	**0.009**
**VLF (0 - 0.04), med (Q1-Q3)**	(28, 48, 49)	88.59 (84.67 - 92.6)	88.49 (84.33 - 92.26)	93.2 (88.21 - 94.53)	**0.011**
**VLF (0.003 - 0.04), med (Q1-Q3)**	(13)	88.59 (84.67 - 92.6)	88.49 (84.33 - 92.26)	93.2 (88.21 - 94.53)	**0.011**
**LLF (0.04 – 0.08), med (Q1-Q3)**	(49)	17.17 (12.37 - 22.85)	17.47 (12.77 - 23.02)	11.42 (7.98 - 17.11)	**0.007**
**LF (0.02-0.14) med (Q1-Q3)**	(51)	53.33 (47.49 - 60.48)	53.61 (47.76 - 60.56)	48.5 (39.27 - 53.18)	**0.018**
**LF (0.03 - 0.07) med (Q1-Q3)**	(47)	49.85 (43.98 - 55.51)	50.34 (44.44 - 55.59)	45.95 (37.57 - 49.41)	**0.019**
**LF (0.03 - 0.15), med (Q1-Q3)**	(3, 31-35, 53, 55)	53.76 (47.87 - 60.86)	54.02 (48 - 61.28)	48.86 (39.6 - 53.69)	**0.018**
**LF (0.03125-0.1) med (Q1-Q3)**	(23)	18.38 (13.18 - 23.94)	18.56 (13.25 - 24.1)	12.12 (8.51 - 18.18)	**0.007**

**LF (0.04 - 0.15), med (Q1-Q3)**	(11, 13, 20, 21, 27, 28, 42, 46, 48, 52, 54, 59, 61)	20.14 (14.3 - 26.54)	20.36 (14.53 - 26.58)	13.34 (9.71 - 20.29)	**0.007**
**LF (0.08 -0.15), med (Q1-Q3)**	(49)	5.46 (3.55 - 7.82)	5.52 (3.71 - 7.94)	3.67 (2.6 - 5.28)	**0.014**
**MF (0.07 -0.13), med (Q1-Q3)**	(47)	7.54 (5.19 - 10.75)	7.65 (5.42 - 10.85)	4.84 (3.48 - 7.93)	**0.010**
**MF (0.1-0.4), med (Q1-Q3)**	(51)	6.2 (3.96 - 8.25)	6.24 (4.12 - 8.32)	4.03 (2.7 - 6.14)	**0.021**
**MF (0.15 - 0.5), med (Q1-Q3)**	(3, 21, 31-35, 46, 51, 55)	3.5 (2.15 - 4.6)	3.57 (2.22 - 4.65)	2.38 (1.49 - 3.67)	**0.036**
**HF (>0.15), med (Q1-Q3)**	(49)	4.15 (2.61 - 5.53)	4.16 (2.71 - 5.56)	2.72 (1.73 - 4.31)	**0.032**
**HF (0.13-1), med (Q1-Q3)**	(47)	4.74 (3.02 - 6.21)	4.75 (3.11 - 6.24)	3.13 (2 - 4.87)	**0.039**
**HF (0.15 - 0.4), med (Q1-Q3)**	(11, 13, 28)	3.32 (2.03 - 4.33)	3.38 (2.06 - 4.41)	2.23 (1.42 - 3.47)	**0.033**
**HF (0.15 - 1.0), med (Q1-Q3)**	(42, 54, 59)	3.96 (2.49 - 5.25)	3.98 (2.57 - 5.27)	2.62 (1.66 - 4.13)	**0.036**
**HF (0.4 - 1.5), med (Q1-Q3)**	(20, 27, 48, 52)	0.95 (0.58 - 1.34)	0.95 (0.6 - 1.35)	0.57 (0.36 - 0.95)	**0.038**
**HF (0.4 - 1.4), med (Q1-Q3)**	(51)	0.92 (0.56 - 1.3)	0.92 (0.58 - 1.3)	0.55 (0.35 - 0.93)	**0.041**
**HF (0.5 - 1), med (Q1-Q3)**	(3, 21, 31-35, 46, 53, 55)	0.47 (0.28 - 0.68)	0.48 (0.29 - 0.68)	0.28 (0.17 - 0.49)	**0.043**
**VHF (0.75 - 1.5), med (Q1-Q3)**	(28)	0.38 (0.23 - 0.51)	0.39 (0.24 - 0.52)	0.22 (0.15 - 0.35)	**0.018**

Bold: significant p-values. Abbreviations: VLF, very low frequency; LLF, low low frequency; LF, low frequency; MF, movement frequency; HF, high frequency; VHF, very high frequency; med, median; Q1, first quartile; Q3, third quartile.

Table S4 - Comparison of spectral bands energy between non-acidemic (n=239) and acidemic fetuses (n=7), for a pH cutoff of 7.05

Frequency Band (Hz)	References	Total	Non-acidemic	Acidemic	Mann-Whitney p-value
**VLF (0 - 0.03), med (Q1-Q3)**	(3, 31-35)	84.25 (79.19 - 89.82)	84.24 (79.03 - 89.72)	89.62 (82.48 - 95.37)	**0.084**
**VLF (0 - 0.04), med (Q1-Q3)**	(28, 48, 49)	88.59 (84.67 - 92.6)	88.56 (84.53 - 92.59)	92.22 (86.75 - 97.05)	0.128
**VLF (0.003 - 0.04), med (Q1-Q3)**	(13)	88.59 (84.67 - 92.6)	88.56 (84.53 - 92.59)	92.22 (86.75 - 97.05)	0.128
**LLF (0.04 – 0.08), med (Q1-Q3)**	(49)	17.17 (12.37 - 22.85)	17.39 (12.7 - 23.01)	11.76 (6.75 - 16.73)	**0.020**
**LF (0.02-0.14) med (Q1-Q3)**	(51)	53.33 (47.49 - 60.48)	53.35 (47.76 - 60.56)	42.06 (37.74 - 53.51)	**0.018**
**LF (0.03 - 0.07) med (Q1-Q3)**	(47)	49.85 (43.98 - 55.51)	50.22 (44.44 - 55.59)	39.6 (36.18 - 49.84)	**0.015**
**LF (0.03 - 0.15), med (Q1-Q3)**	(3, 23, 31-35, 55)	53.76 (47.87 - 60.86)	53.8 (48 - 61.28)	42.3 (37.84 - 54.05)	**0.018**
**LF (0.03125-0.1) med (Q1-Q3)**	(23)	18.38 (13.18 - 23.94)	18.46 (13.21 - 24.09)	12.41 (6.93 - 17.98)	**0.019**
**LF (0.04 - 0.15), med (Q1-Q3)**	(11, 13, 20, 21, 27, 28, 42, 48, 52, 54, 59, 61)	20.14 (14.3 - 26.54)	20.28 (14.35 - 26.57)	13.62 (7.43 - 19.86)	**0.020**
**LF (0.08 -0.15), med (Q1-Q3)**	(49)	5.46 (3.55 - 7.82)	5.48 (3.55 - 7.84)	3.96 (1.45 - 6.64)	0.114
**MF (0.07 -0.13), med (Q1-Q3)**	(47)	7.54 (5.19 - 10.75)	7.58 (5.21 - 10.83)	5.76 (2.13 - 8.4)	0.124
**MF (0.1-0.4), med (Q1-Q3)**	(51)	6.2 (3.96 - 8.25)	6.2 (3.99 - 8.29)	3.89 (1.46 - 7.45)	0.125
**MF (0.15 - 0.5), med (Q1-Q3)**	(3, 21, 31-35, 46, 55)	3.5 (2.15 - 4.6)	3.51 (2.22 - 4.63)	1.91 (0.8 - 4.4)	0.132

**HF (>0.15), med (Q1-Q3)**	(49)	4.15 (2.61 - 5.53)	4.15 (2.7 - 5.54)	2.3 (0.97 - 5.42)	0.154
**HF (0.13-1), med (Q1-Q3)**	(47)	4.74 (3.02 - 6.21)	4.75 (3.05 - 6.21)	2.79 (1.14 - 6.13)	0.162
**HF (0.15 - 0.4), med (Q1-Q3)**	(11, 13, 28)	3.32 (2.03 - 4.33)	3.34 (2.06 - 4.35)	1.79 (0.77 - 4.01)	0.115
**HF (0.15 - 1.0), med (Q1-Q3)**	(42, 54, 59)	3.96 (2.49 - 5.25)	3.96 (2.53 - 5.26)	2.18 (0.92 - 5.13)	0.156
**HF (0.4 - 1.5), med (Q1-Q3)**	(20, 27, 48, 52)	0.95 (0.58 - 1.34)	0.95 (0.59 - 1.34)	0.57 (0.22 - 1.53)	0.312
**HF (0.4 - 1.4), med (Q1-Q3)**	(51)	0.92 (0.56 - 1.3)	0.92 (0.57 - 1.3)	0.56 (0.21 - 1.45)	0.315
**HF (0.5 - 1), med (Q1-Q3)**	(3, 21, 31-35, 46, 55) (35)	0.47 (0.28 - 0.68)	0.47 (0.28 - 0.68)	0.29 (0.12 - 0.66)	0.287
**VHF (0.75 - 1.5), med (Q1-Q3)**	(28)	0.38 (0.23 - 0.51)	0.38 (0.23 - 0.51)	0.22 (0.09 - 0.5)	0.239

Bold: significant p-values. Abbreviations: VLF, very low frequency; LLF, low low frequency; LF, low frequency; MF, movement frequency; HF, high frequency; VHF, very high frequency; med, median; Q1, first quartile; Q3, third quartile.
